# Interdisciplinary aspects of abdominal and plastic surgery – what does the (abdominal) surgeon need to know?

**DOI:** 10.1515/iss-2023-0042

**Published:** 2023-09-21

**Authors:** Armin Kraus, Hans-Georg Damert, Frank Meyer

**Affiliations:** Department of Plastic, Aesthetic and Hand Surgery, Otto von Guericke University at Magdeburg with University Hospital, Magdeburg, Germany; Department of Plastic, Aesthetic and Hand Surgery, Regional Hospital (Helios-Bördeklinik), Oschersleben/Neindorf, Germany; Department of General, Abdominal, Vascular and Transplant Surgery, Otto von Guericke University at Magdeburg with University Hospital, Magdeburg, Germany

**Keywords:** plastic surgery, abdominal surgery, pediculated/free flap transfer, rectus-abdominis flap, latissimus-dorsi flap

## Abstract

**Introduction:**

The aim was to reflect the established interdisciplinary aspects of general/abdominal and plastic surgery by means of a narrative review. Methods: (i) With specific references out of the medical literature and (ii) own clinical and perioperative as well as operating technical and tactical management experiences obtained in surgical daily practice, we present a choice of options for interdisciplinary cooperation that could be food of thought for other surgeons.

**Content:**

– Decubital ulcers require pressure relieve, debridement and plastic surgery coverage, e.g., by a rotation flap plasty, V-Y flap or “tensor-fascia-lata” (TFL) flap depending on localization (sacral/gluteal defects, ischiadic tuber). – Coverage of soft tissue defects, e.g., after lymph node dissection, tumor lesions or disturbance of wound healing can be managed with fasciocutaneous or muscle flaps. – Bariatric surgery: Surgical interventions such as butt lift, tummy tuck should be explained and demonstrated in advance and performed commonly after reduction of the body weight. – Abdominoperineal rectum extirpation (APE): Holm’s procedure with greater circumferential extent of resection at the mesorectum and the insertion site of the levator muscle at the anal sphicter muscle resulting in a substantial defect is covered by myocutaneous flap plasty. – Hernia surgery: Complicated/recurrent hernias or abdominal wall defect can be covered by flap plasty to achieve functional reconstruction, e.g., using innervated muscle. Thus, abdominal wall can respond better onto changes of pressure and tension. – Necrotising fasciitis: Even in case of suspicious fasciitis, an immediate radical debridement must be performed, followed by intensive care with calculated antibiotic treatment; after appropriate stabilization tissue defects can be covered by mesh graft of flap plasty. – Soft tissue tumor lesions cannot be resected with primary closure to achieve appropriate as intended R0 resection status by means of local radical resection all the time – plastic surgery expertise has to be included into interdisciplinary tumor concepts. – Liposuction/-filling: Liposuction can be used with aesthetic intention after bariatric surgery or for lipedema. Lipofilling is possible for reconstruction and for aesthetic purpose. – Reconstruction of lymphatic vessels: Lymphedema after tumor operations interrupting or blocking lymphatic drainage can be treated with microsurgical reconstructions (such as lympho-venous anastomoses, lympho-lymphatic anastomoses or free microvascular lymph node transfer). – Microsurgery: It is substantial part of modern reconstructive plastic surgery, i.e., surgery of peripheral nerves belongs to this field. For visceral surgery, it can become important for reconstruction of the recurrent laryngeal nerve. – Sternum osteomyelitis: Radical debridement (eventually, complete sternal resection) with conditioning of the wound by vacuum-assisted closure followed by plastic surgery coverage can prevent chronification, threatening mediastinitis, persisting infectious risk, long-term suffering or limited quality of life.

**Summary:**

The presented selection of single topics can only be an excerpt of all the options for surgical cooperation in daily clinical and surgical practice.

**Outlook:**

An interdisciplinary approach of abdominal and plastic surgery is characterized by a highly developed cooperation in common surgical interventions including various techniques and tactics highlighting the specifics of the two fields.

## Introduction

Plastic and abdominal surgery have developed rapidly. In this context, there are numerous subject-specific areas of activities and research. Particularly, if large defect occur after tumor resection of wound debridement, plastic surgery can assist both in defect closure and in reconstruction of form and function. As every reconstruction is also an issue of aesthetics, plastic surgery can help to increase patient satisfaction by creating a result that is not only functional, but also acceptable in social interaction and activities of daily living. Therefore, patient quality of life can be improved.

Plastic surgery offers a variety of techniques therefor: nerval transplantation, lymphangioreconstruction, lymph node transfer, free tissue transfer, [re-]transplantation of extremities, facial transplantation etc. are some of the cutting edge achievements of plastic surgery. Although not all of these are performed in daily routine, several tools of plastics can be beneficial in cooperation with abdominal surgery, and some of these will be illustrated further on. A short outlook will although be given on academic and basic science cooperation, and their possible future developments.

The *aim* of this compact short overview is based on

(i) specific references out of the medical literature (and)
(ii) own clinical and perioperative as well as operating technical and tactical management experiences obtained in surgical daily practice
  to illustrate the routinely established interdisciplinary aspects between abdominal and plastic surgery and to teach young graduates of the study of human medicine who decide for (general/abdominal) surgery as well as to remind experienced colleagues with an up-dated version in terms of “What does the (general/abdominal) surgeon need to know?”.

## Corner points

### Overlapping diagnoses în the spectrum of care of the disciplines

Within the spectrum of care of plastic surgery on one side as well as general and abdominal surgery on the other side, there is overlap in numerous diagnoses, e.g.,

– surgery of the body surface with small tumor lesions of the skin,

### Lymph node excision/-dissections (or even)

– excision of lipomas at various sites.

The disciplines have increasingly specialized over the last decades and each specialty has its own working field but in several diagnoses, activities and care overlap, e.g., in the

– treatment of hernias, in particular, recurrent hernias,
– reconstruction of the abdominal wall (and)
– coverage of tissue defects after previous abdomino- or vascular surgical interventions.

In the area of bariatric surgery, there is also a close and effective cooperation of the disciplines. The field of expertise of plastic surgery focusses onto reconstruction of tissue defects with “autologous material”, more rarely using meshes. Thus, it is possible to cover tissue defetcs sufficiently using flap plasties in case of necessary explantation of meshes after complex abdomino surgical interventions of previous hernias or defects of the anterior abdominal wall with complicated postoperative courses. If larger defects or defect wounds are the consequences of the treatment of malignant soft tissue tumors or in case of necrotizing fasciitis, an interdisciplinary collaboration is desirable and partially even required to perform a sufficient coverage of tissue defects. In this context, it is helpful to go for an interdisciplinary approach prior to the primary surgical procedure.

### Most frequent service operation of plastic surgery

#### Decubital ulcer

Surgical treatment of decubital ulcer has developed to one of the leading diagnoses with regard to its frequent occurrence, treatment activities and case numbers, in particular, in more advanced stages, which preferentially need defect coverage by plastic surgery. An early case presentation in or even transferral to plastic surgery seems to be, therefore, recommendable [1, 2]. It often happens that due to fear of large, not manageable tissue defects, prolonged clinical course is provoked [3]. However, this can lead to a longer hospital stay or duration of treatment and decreased quality of life as well as increased treatment costs [4, 5]. Profession collaboration in “Wound Centers”, in particular, within greater hospitals, has been proven successfully. In case of newly diagnosed decubital ulcer, e.g., occurring during a prolonged or complicated hospital stay, consultation of plastic surgery is reasonable [6, 7]. Possibly, sufficient treatment can be already initiated during the same hospital stay or even reliably planned for the near future. Depending of the decubital ulcer site, various flap plasties are available [8–17].

**Figure 1:**
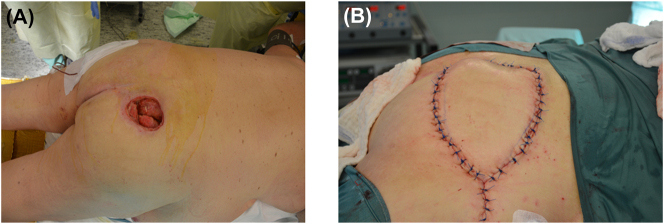
Clinical finding of decubital ulcer pre- and post-operatively. (A) Preoperative situs: substantial skin defect with superficial infection; (B) postoperatively: wound area after local rotation flap plasty.

*Most frequent tissue defects*:

Sacral and gluteal defects (examples):

– Rotation flap plasty (fasciocutaneous, gluteal) – Figure 1,
– V-Y flap plasty (myocutaneous); gluteal uni- or bilateral,
– “Tensor-fascia-lata” flap plasty (TFL);

Tuber ischiadicum (examples):

– V-Y flap plasty (myocutaneous; biceps-femoris flap plasty, Harmstring-flap plasty),
– TFL-flap plasty.

#### Coverage of soft tissue defects

**Figure 2:**
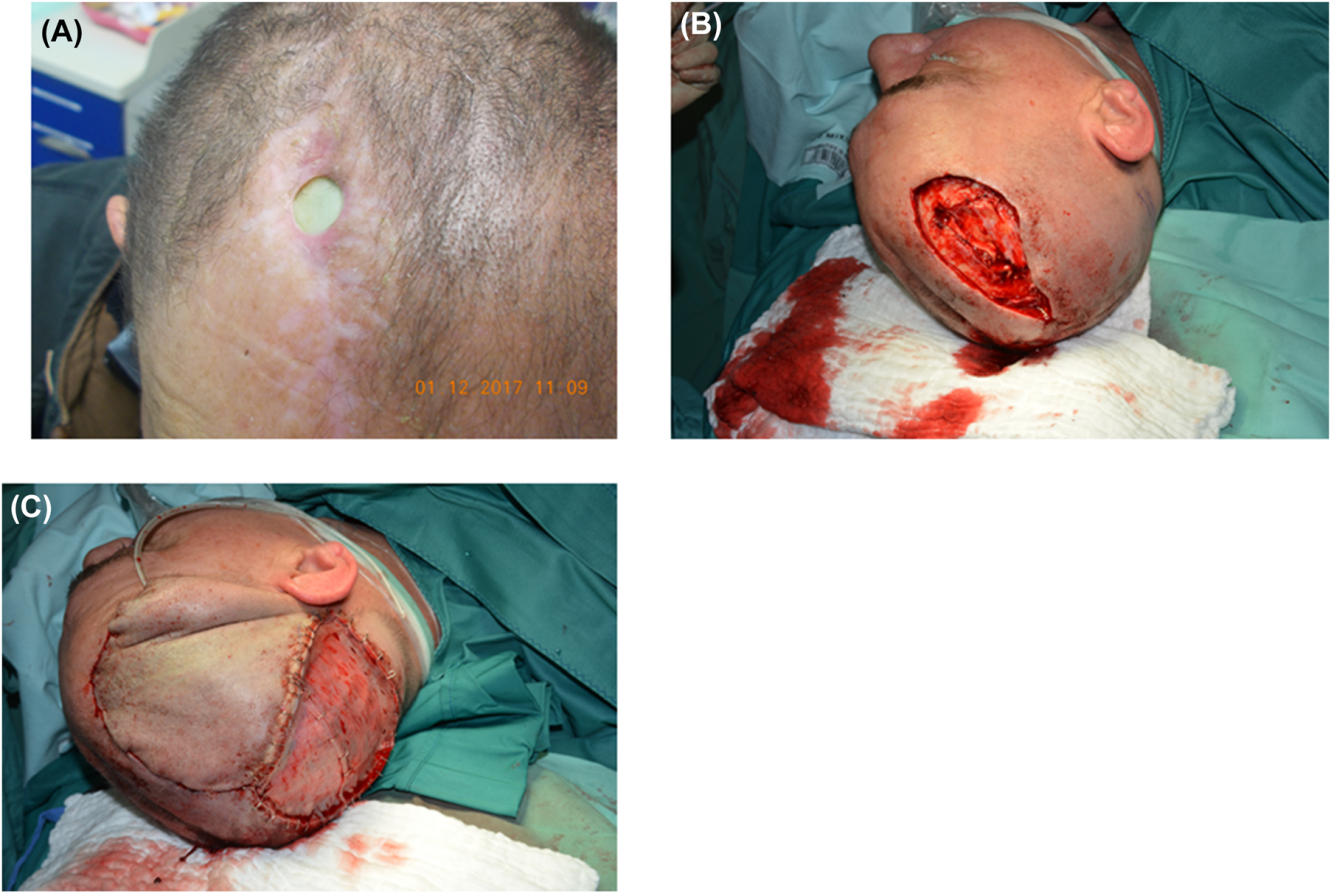
Infected wound area of the right forehead. (A) Preoperative infected wound with skin defect; (B) intraoperative wound sites after extensive excision; (C) at the end of the surgical intervention: rotation flap plasty with coverage of the fresh skin defect with mesh graft.

**Figure 3:**
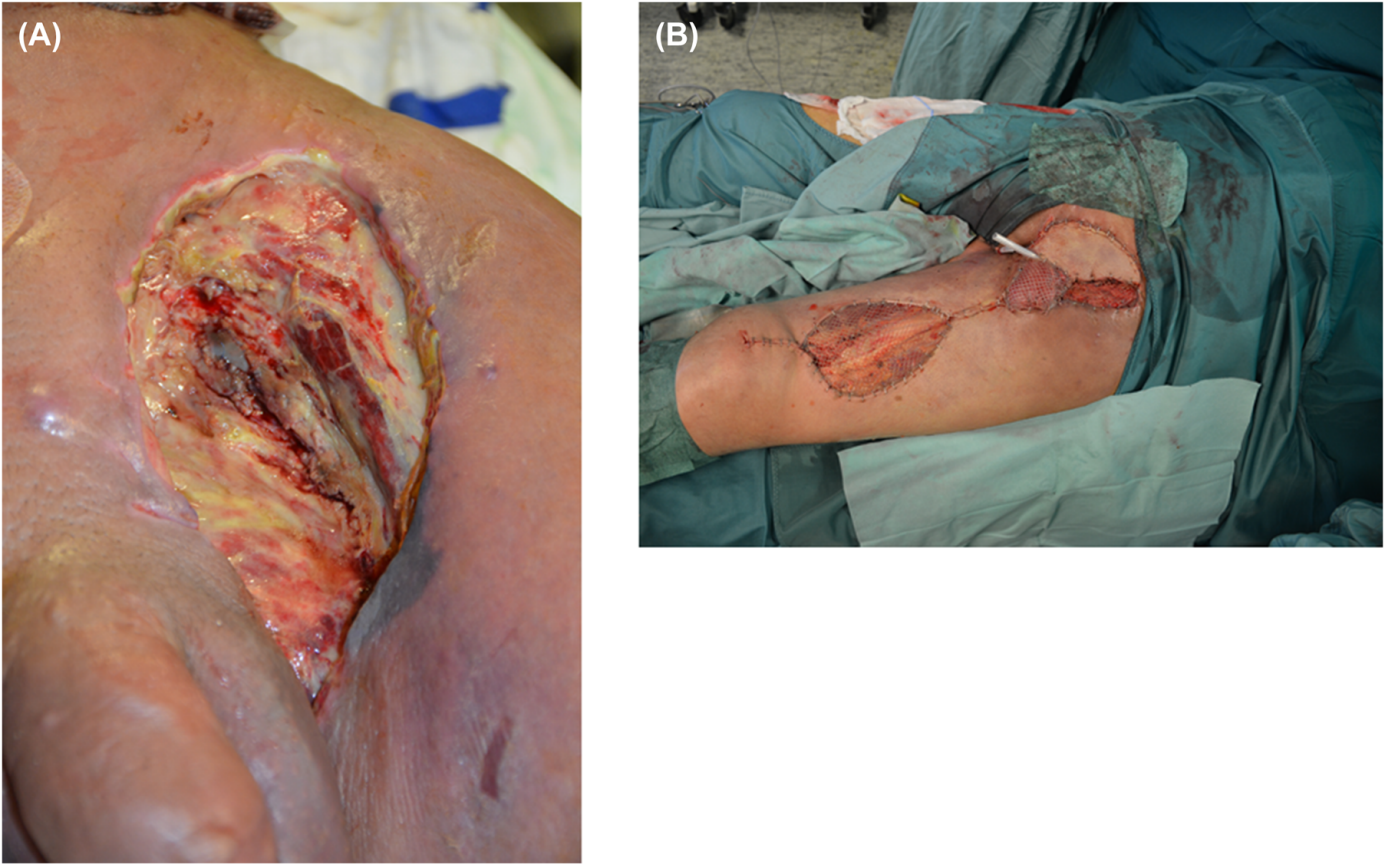
Infected wound due to infected vascular prosthesis. (A) Infected wound area at the left groin prior to surgical revision; (B) coverage of the excized wound with “Gracilis-muscle” plasty (tissue transferral to the left groin and coverage of the left distal thigh with mesh graft).

They may become required, e.g.,

– after lymph node dissections,
– in tumor lesions
– in case of disturbed wound healing

after primary care by general or abdominal surgeons. The coverage of soft tissue can be either considered a “service” of plastic surgery. The basic disease is not treated in a sufficient manner but there is still a remaining defect, e.g., after extended lymph node dissections, persistent disturbance of wound healing may result occasionally associated with chronic fistulas. In such case, muscle flap plasty may be considered a good approach. After vascular surgical interventions with subsequent disturbed wound healing and open wound area with vascular prosthesis or venous bypass in the groin, plastic surgery for coverage of the wound area, e.g., by means of a fasciocutaneous or muscle flap plasty (TFL, rectus femoris, vastus medialis or lateralis) may preserve the extremity [18–20] (Figures 2 and 3).

#### Lymphatic vessel reconstructions

Tumor operations associated with the interruption or blockage of the lymphatic drainage can result in formation of lymphedema. The same can happen after radiation therapy (secondary lymphedema). Lymphtic vessels might be well developed congenitally (primary lymphedema). In both cases, there is an altered drainage of chyle leading to a chronic swelling and increased protein deposition within the tissue. This can lead to a low quality of life. Prior to the development of microsurgery, it was only possible to mitigate symptoms by conservative measures such as complex manual decongestion therapy. Using microsurgical techniques and its further development make it possible to reconstruct impaired lymphatic vessels to improve, thus, lymphatic transport. While lymphovenous anastomoses have been already created in suitable indications for many years, the free microvascular lymph node transfer has been used with better results in a few centers over the last years. For instance, after radical axillary dissection lymphedema of arms can be improved using free lymph node transfer from a healthy body part. In addition, transplanted lymph nodes are immuncompetent with the consequence that there is a lower frequency of infections.

#### Bariatric surgery

As substantial part of bariatric operations, cooperation of general/abdominal surgery and plastic surgery has been established for years. Thus, patients can be notified on the treatment (e.g., “gastric sleeve resection”) including possible surgical interventions provided by plastic surgery resecting fatty or connective tissue after substantial loss of body weight. The close collaboration of the disciplines in “Obesity Centers” guarantees short ways for the patients and accompanies them after weight loss. Even for other medical disciplines such as gynecology, urology, plastic surgeons provide “service operations” in morbidly obese patients [20–22]. For example, resection of pendulous abdomen as part of abdominosurgical interventions can facilitate access and overview as well as it can promote postoperative healing.

#### Abdominoperineal rectum extirpation (APE)

The cylindric APE (abdominoperineal rectum extirpation) according to Holm’s procedure has led to an oncological advantage of rectal cancer with regard to the prognosis versus conventional APE [23–25]. This comprises the extralevatory resection of low rectal cancer providing – in contrast to conventional APE – an extended circumferential resection at the mesorectum and the insertion of the levatory muscle at the sphincter muscle. Frequently, disturbances of perineal wound healing can occur, which can be a big challenge for the postoperative wound management [26]. In addition, such chronic and sometimes secretive wounds can become a heavy burden for the patient and her/his relatives. The closure of resulting defects using a myocutaneous flap plasty has proved successful [27]. According to the extent of the defect, various flap plasties are considered [23, 24, 28–35]. While smaller defects can be covered with myocutaneous gluteal flap plasties or simple gracilis-muscle flap plasties, the VRAM-flap plasty (“Vertical Rectus-Abdominis-Muscle” flap plasty) for large perineal defects. This comprises – as the flap desgination already indicates – that a rectus-abdominus muscle including a superficial skin part is transferred via a transpelvine route to the defect. Thus,

– the tissue defect can be filled with muscle and skin defect can be sufficiently closed to outside, and
– by the muscle transfer, a sufficient blood supply can be provided in the target area.

**Figure 4:**
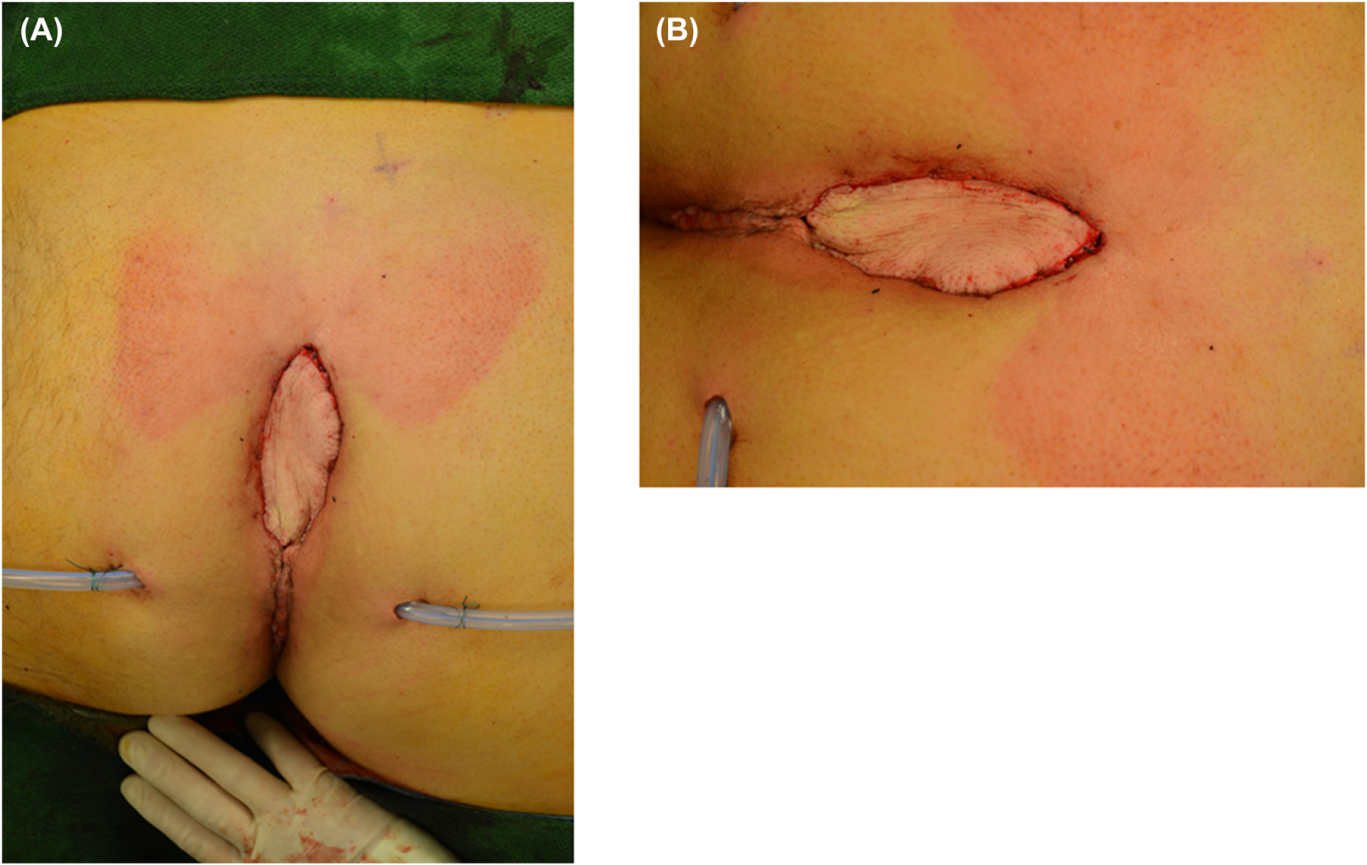
Postoperative (formally perianal) wound area after Holm’s procedure for rectal cancer and “Rectal-muscle” plasty. (A) View onto the postoperative wound area – lithotomy position; (B) view onto the postoperative wound area of the patient laying on the bed.

This can substantially improve the local situation, in particular, after neoadjuvant radiochemotherapy (Figure 4).

#### Hernia surgery

Hernia treatment is one of the domains of general and abdominal surgery. This comprises open surgical techniques as well as minimally invasive techniques. In particular, various types of adapted meshes are used [36]. In complicated courses with infected meshes or large defects of the abdominal wall, plastic surgery can be helpful. Frequently, complicated recurrent hernias or defects of the abdominal wall can be covered by own tissue using flap plasties [37, 38, 39–41]. Such approach is superior to a “simple” mesh graft, e.g., directly on the intestine or mesh. On one hand, a better stability and coverage of the tissue defect can be achieved and, on the other hand, with regard to later surgical interventions, there will be a lower risk for potential intestinal alterations. Compound separation according to Ramirez can be performed both by abdominal or plastic surgeons, depending on individual skills.

By a functional reconstruction of abdominal wall defects, e.g., using innervated muscle, abdominal wall is sufficiently resistant to pressure and tension. For defects of the lower abdominal wall, a pediculated rectus-femoris-muscle flap plasty is recommendable [38, 40–43], particularly since morbidity of the flap mobilization is low. If a pediculated flap plasty in the required area cannot be performed, also free flap plasties are available, which can be used according to the defect site.

#### Necrotizing fasciitis

Necrotizing fasciitis (NF) is defined as a rare and life-threatening infection of subcutaneous tissue and fascia [44] as well as a fast progression of the disease associated with severe pain, which cannot be referred to the initially moderate clinical finding. Since laboratory parameters as well as imaging are unspecific, diagnosis finding is pursued with clinical issues. Already the suspicion requires a prompt radical debridement, in the majority of cases, repeatedly added by intensive care and antibiotic therapy. After stabilization of general condition and wound situation, coverage of defects and reconstruction by plastic surgery techniques is sometimes required [18, 37, 40, 45]. This includes mesh grafting, but also muscle or myocutaneous flaps. The fear of large defects as consequence of radical surgery can result in fatal outcome. Under such circumstances, a plastic surgeon needs to be involved early. For genital region, collaboration with the urologist can be helpful.

#### Soft tissue tumor lesions

In the treatment of soft tissue tumor lesions, there is overlap of several surgical disciplines, such as abdominal surgery, maxillary surgery, otorhinolaryngology, neurosurgery, orthopedic surgery etc. There are benign and malignant tumor lesions at each site of the body [19, 20]. In particular, in malignant or locally aggressive tumor lesions, primary wound closure is not always possible to achieve R0-resection status. If radical or extensive surgery is substantial for an appropriate therapy and prognosis, fear of a large tissue defect should not hinder the surgeon to achieve appropriate radicality. In this context, interdisciplinary tumor board conference is a suitable forum to derive the best tumor- and patient-associated therapy.

#### Liposuction

Liposuction can be considered rather a domain of plastic surgery. However, it is not only used for aesthetic purpose [46, 47–51]; it can become part

– of various disciplines,
– bariatric operations,
– in lipedema (and)
– for reconstruction (e.g., “lipofilling”).

A novel approach using liposuction – mainly in obese patients – is also possible in shunt surgery. Therein, deep vessels, in particular, veins can be elevated by liposuction closer to the surface with no need to dissect the vessel [52].

#### Lipofilling

Over the last years, treatment with own fatty tissue has been established, in particular, in plastic surgery [51, 53–55]. There is an option to use endogeneous “material”. In addition, the spectrum of indications has been distinctly extended. Variably processed fatty tissue can be used for several indications:

– rhizarthrosis (intraarticular injection),
– wrinkle treatment with own fatty tissue (instead of hyaluronic acid),
– treatment of scars,
– breast augmentation (aesthetic and for reconstruction),
– treatment of chronic ulcers.

#### Sternum osteomyelitis

Sternum osteomyelitis – can be relevant even in abdominal surgery. It occurs after sternotomy, e.g., as part of bypass operations, after other thoracic surgery interventions or after trauma. It can be considered a severe complication with a mortality up to 30 % according to the literature [56]. In addition to the exogeneous spread, the endogeneous spread can also become life-threatening, and may lead to a fulminant mediastinitis. Therefore, the aim should be to sanitize the infection including a sufficient plastic-surgery coverage of the defect [18, 34]. An insufficient radical debridement can lead to a prolonged hospital stay with possible chronification, which can mean lifelong suffering, a persistent infectious risk and a reduced quality of life. Furthermore, there will be increased costs by the long duration of treatment.

**Figure 5:**
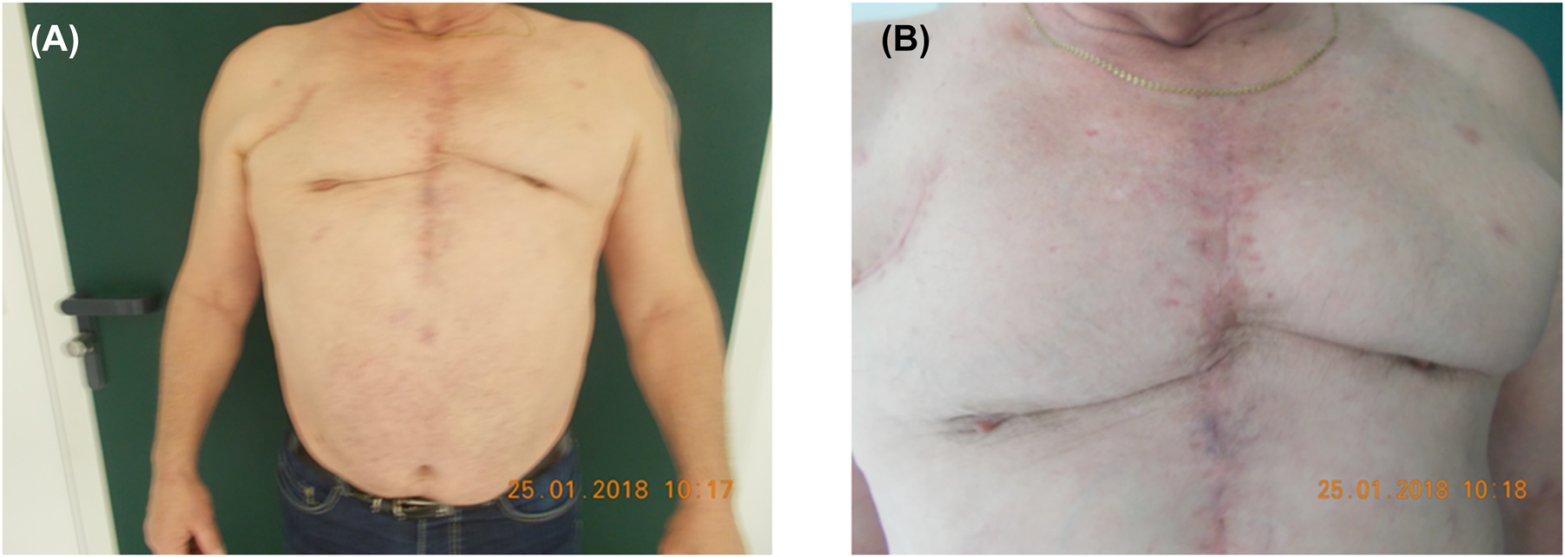
Postoperative results after plastic surgery of sternum osteomyelitis. (A) From distance; (B) near the scar.

Occasionally, complete removal of the sternum is necessary. In the meantime, wound conditioning can be performed using vacuum-assisted closure. The early consultation of a plastic surgeon should be considered. Knowing the possible options of coverage of tissue defects, inhibition level might be lower in performing radical debridement (Figure 5).

To cover tissue defects, the following flap plasties are available if a primary woud closure is not possible:

– Pectoral-muscle flap (pediculated; unilateral or bilateral),
– Latissimus-dorsi flap (pediculated; with skin insula),
– VRAM (Vertical Rectus-Abdominis-Myocutaneous flap),
– Free flap plasties.

Table 1 summarizes the suggestions for interdisciplinary treatment of the pathologies described above.

**Table 1:** Summary of potential treatment approaches in common clinical findings that require interdisciplinary cooperation.

Underlying clinical problem	Suggested interdisciplinary treatment
Decubital ulcer	Various kinds of local and regional fasciocutenous and muscle flaps (mainly gluteus, hamstring, tensor fasciae latae)
Soft tissue defects or fistulas (lymp node dissection, tumor resection, impaired wound healing)	Depending on the body region - skin grafts, local or regional fasciocutaneous or muscle flaps, free flaps in selected cases, lipofilling
Obesity and massive weight loss after bariatric surgery	Tummy tuck, brachioplasty and genioplasty, contouring liposuction
Pelvic floor defects after APE	VRAM flap
Hernia surgery	Innervated muscle flaps, regional flaps from the upper leg
Necrotizing fasciitis	Split thickness skin grafts after radical debridement, local or free flaps if required
Soft tissue tumor lesions	Skin grafts, local, regional or free flaps
Lipedema	Liposuction
Lymphedema	Lymph vessel reconstruction or lymph node transplantation
Sternum osteomyelitis	Pedicled muscle flaps (pectoralis, latissimus, VRAM), free flaps in selected cases

APE, abdominoperineal rectum extirpation; VRAM, vertical rectus adominis myocutaneous/muscle flap.

### Common teaching concepts

The medical and, in particular, the surgical teaching is increasingly considered as an

– interdisciplinary,
– multimedial (and)
– interactive

approach associated with OSCE-relevance in lectures, seminars, medical clerkships, block internships or facultative teaching events, even in exams.

In this context, surgical disciplines appear suitable to generate a complementary holistic teaching strategy due to coordinated concepts and to develop cooperative collaboration based on similar thinking, approach and action.

Based on the several points of contact, the collaboration of plastic and general/abdominal surgery has a pioneer role (e.g., clinical course in “surgical suturing”).

### Study/research activities

The avove mentioned overlaps of both surgical disciplines are also an important reason for common research activities. In clinical research, in particular, hernia aspects can be interdisciplinarily assessed, e.g., with regard to reconstruction details. The same is possible in greater oncosurgical interventions, which need coverage of a defect. Even in basic research,

– “Tissue Engineering” (cell culture, cell transfer etc.),
– Stem cells (or)
– Cancer research.

Fields such as treatment of sarcomas of the extremities, tissue engineering and sepsis pathophysiology are suitable topics for interdisciplinary research activities of plastic and general/abdominal surgery.

### New aspects & trends

Over the last years, plastic surgery has developed. One part of this is to the treatment with own fatty tissue [54] not only for aesthetic indications but also as part of treatment modalities for wounds and scars as well as for breast reconstruction. Withdrawn fatty tissue can be variably conditioned for several indications before it will be transplanted as autologous cell/tissue transfer. Hot topic of current discussions and subject of further investigations is the function and consequence of the contained and also transferred stem cells as well as the question whether processed probes underly the “Medicinal product law” (in German, “Medizinproduktegesetz”). In this context, further developments are expected within the next years. The preparation of guidelines for autologous fat transplantation has to provide more therapeutic safety. Furthermore, stem cells might facilitate wound healing, as well as the addition or inhibition of certain mediators, such as VEGF or EGF optionally delivered by extracellular vesicles (EVs). With regard to the therapy of lymphedema, further studies need to show whether microsurgical techniques (such as free lymph node transfer) may be able to provide a permanent alternative in the related indications. This is, in particular, desirable for breast cancer. Some of these cases will surpass human manual abilities, so that robotic surgery could be a remedy. First studies have shown promising results, but robotics has not become a routine tool in plastic surgery so far.

Drug therapy of “chronic inflammation” in lymphedema is currently subject of research [39]. It aims at reducing chronic irritation, which may lead to fibrotization and other pathological tissue effects. However, it will most likely take several years to the development of an effective drug and its subsequent approval.

### Academic activities

On an academic level, there are quite a few synergies, which can be used for the interdisciplinary cooperation of abdominal and plastic surgery. In academic councils, such as teaching commissions, ethic committee or the “Commission to Promote Scientific Junior Staff”, it should be seriously attempted to strengthen the common surgical interests. This would be beneficial for research and teaching of the surgical discipline as well as keep surgery sustainable. Commonly supervised dissertations (M.D. thesis) at the interfaces of abdominal and plastic surgery or even Ph.D. thesis would also strengthen both disciplines on an academic level.

### Limitations (and perhaps strengths) of this review

Although written to the authors’ best knowledge, the work has several limitations that should be addressed. As this article is an attempt to present the overlapping fields of abdominal and plastic surgery in its broad variety, the cited literature is selected, but not comprehensive. We encourage other authors to take this work as some food of thought for the writing of more detailed articles on the sub-sections as were depicted here. Furthermore, this work was intended as a clinical review, so that the focus was not on basic science concepts, although briefly included. Overall, the fact that all procedures presented in this work have been applied by the authors for several years, and their description and evaluation has been made from a practical and honest point of view, may give some value to this article.

## Conclusions

An interdisciplinary approach of different medical specialties, in particular, of surgical subdisciplines, cannot be assumed away from the challenging daily clinical practice, e.g., in takeover of subsequent subtasks or in the formation of interdisciplinary surgical teams. In particular, this includes surgical care of complex oncosurgical diseases, such as tumor manifestation within the pelvis (e.g. rectal cancer or sarcoma). By the ongoing specialization of the various fields and the advancement of surgical procedures, especially of the microsurgical techniques, interdisciplinary treatment concepts and strategies are recommendable. It should be a simple task to consult an expert from a flanking surgical discipline for treatment or to transfer the patient into colleagues‘ hands. Patients’ optimal treatment should be always in the front.

## Supplementary Material

Supplementary Material
