# Supplementary material for: Interdisciplinary aspects of abdominal and plastic surgery – what does the (abdominal) surgeon need to know?
Source: Innov Surg Sci. 2023 Sep 21;8(2):103–12. doi: 10.1515/iss-2023-0042 (PMC10696945; doi:10.1515/iss-2023-0042)
Supplement: Supplementary file 1 — Supplementary Material [file j_iss-2023-0042_suppl_001.pdf]

Reviewer Assessment

A. Kraus, H-G. Damert & F. Meyer: Interdisciplinary aspects of abdominal and plastic surgery – what does the (abdominal) surgeon needs to know?

Reviewers' Comments to Original Submission

Reviewer 1: Jannasch, Olof

Date received: 09-Jun-2023
Reviewer recommendation: **Return to author for minor modifications**
Reviewer overall scoring: **Medium**

Comments to author:
Points for modification:
Abstract - the method is missing, the results are to long and need to be restructured and new aspects need to highlighted
Introduction - it should be more related to the following report
Coverage of soft tissue defects - it reports of chronic fistulas after lymph node dissection - so besides coverage of the soft tissue defect lymphangioreconstruction should be mentioned
Hernia surgery - Component separation (Ramirenz) should be included
References - have to be checked, Ref. 36 is without year and pages, 4 references are older than year 2000, no references after 2018 are included.
Overall, new aspects of this important topic are hard to find in the paper and should be added by a current literature search.
The written english language has to be improved.

Reviewer 2: anonymous

Date received: 09-Jun-2023
Reviewer recommendation: **Return to author for minor modifications**
Reviewer overall scoring: **High**

Comments to author:
The authors submitted an interesting narrative review on the clinically relevant topic
"What the abdominal surgeon needs to know on (the options and perspectives) of plastic surgery", with an appropriate length and number of words (as well as characters) including instructive figures representing decubital ulcer, wound healing problems, infected wounds (by osteomyelitis, by vascular prostheses) or tissue flap in Holm's procedure (diagnosis: rectal cancer) - in this context, for a balanced proportion of figures vs. tables, is there any data (e.g., from the references), which can be presented by an additional table in a compact manner?

In detail,
- The "Title" is instructive.

- "Abstract" - no query

- "Introduction" characterizes appropriately the reason for this topic and writing it up.

- The "Results" subtitled with "Corner points" subclassifies several interesting topics such as "Decubital ulcer" / "Coverage of soft tissue defects", "Bariatric surgery", "Abdominoperineal rectum extirpation (APE)", "Hernia surgery", "Necrotizing fasciitis", "Soft tissue tumor lesions", "Liposuction", "Lipofilling", "Lymphatic vessel reconstructions" and "Osteomyelitis", which comprise the clinically relevant Problems in daily surgical practice of plastic and abdominal surgery - a fascinating spectrum of single topics all of them with a direct or mediate association to or among the two surgical disciplines.
Interestingly, "New aspects and trends" as well as "Studies / Research" plus "Academic activities"- aspects, which are often underestimated and disvalued - were included in the representative spectrum of topics.

- The "Conclusion" summarizes and evaluates the described aspects - no comments opr remarks.

- “References“: n = 54 (appropriate number)
In particular, please accomplish:
36. Orhan E Efficacy of Liposuction as a Delay Method for Improving Flap Survival
48. Vergleich des Regenerativen Zytokinprofils von Adipose Derived Stromal Cells (ASCs) Gewonnen Mittels Abdominoplastik, Suction Assisted Liposuction (SAL) und Ultrasound Assisted Liposuction (UAL)
with the required bibliographic data!

- “Figures“: - see above (1st sentence)

From the reviewer`s perspective, congratulations to the great idea of the topic and the magnificent workup of the single (sub)topics - the only remaining aspects, which appear reasonable to be added, are:
- What are the particular strengths and limitations of the well readable review as it is usually mentioned at the end of the manuscript right before the “Resumé“?

Based on the aspects as raised, the reviewer concludes to assess the Manuscript with

“Accepted for publication with minor revisions“.

Authors’ Response to Reviewer Comments

Date received: 23-Jun-2023

Reviewer 1:

1) Reviewer's 1st critic was: Abstract - the method is missing, the results are too long and need to be restructured and new aspects need to highlighted

RESPONSE: A method section has been included, the results section was shortened, restructured and new aspects were highlighted by underlining

2) Secondly, the reviewer mentioned: Introduction - it should be more related to the following report

RESPONSE: The introduction was re-written with more relation to the following text.

3) Subsequently, the reviewer raised: Coverage of soft tissue defects - it reports of chronic fistulas after lymph node dissection - so besides coverage of the soft tissue defect lymphangioreconstruction should be mentioned

RESPONSE: A section named “Lymphatic vessel reconstructions” has been included

4) Next, the reviewer's suggestion was: Hernia surgery - Component separation (Ramirez) should be included

RESPONSE: A respective section has been included on page 9.

5) The following comment of the reviewer comprised: References - have to be checked, Ref. 36 is without year and pages, 4 references are older than year 2000, no references after 2018 are included.

Overall, new aspects of this important topic are hard to find in the paper and should be added by a current literature search.

RESPONSE: Ref. 36 has been corrected, and several more up-to-date articles have been cited.

6) The reviewer criticized: The written English language has to be improved.

RESPONSE: Language and style have been improved.

Reviewer 2:

I) First, this reviewer suggested: ... - in this context, for a balanced proportion of figures vs. tables, is there any data (e.g., from the references), which can be presented by an additional table in a compact manner?

RESPONSE: Good point, we added the respective information in "Table 1".

III) The reviewer demanded:

- "References": n = 54 (appropriate number)

In particular, please accomplish:

36. Orhan E Efficacy of Liposuction as a Delay Method for Improving Flap Survival

48. Vergleich des Regenerativen Zytokinprofils von Adipose Derived Stromal Cells (ASCs)

Gewonnen Mittels Abdominoplastik, Suction Assisted Liposuction (SAL) und Ultrasound

Assisted Liposuction (UAL)

with the required bibliographic data!

RESPONSE: We apologize for this flaw and corrected the citations mentioned

III) Finally, the reviewer asked, in particular:

- What are the particular strengths and limitations of the well readable review as it is usually mentioned at the end of the manuscript right before the "Resumé"?

RESPONSE: Good point, we added a corresponding section right before our conclusion - see there.

Many thanks for the recommendation.

The additional text / paragraph is as follows:

" … strengthen both sudisciplines on the an academic level.

Limitations (and perhaps strengths) of this review
Altough written to our best knowledge, our work has several limitations that should be addressed. As this article is an attempt to present the overlapping fields of abdominal and plastic surgery in its broad variety, the cited literature is selected, bu not comprehensive. We encourage other authors to take this work as some food of thought for the writing of more detailed articles on the sub-sections we depicted here. Futhermore, this work was indended as a clinical review, so that our focus was not on basic science concepts, although briefly included. Overall, the fact that all procedures presented in this work have been applied by the authors for several years, and their description and evaluation has been made from a practical and honest point of view, may give some value to this article.

Conclusion

An interdisciplinary approach of … "

As far as it seemed necessary to us, remaining spelling mistakes have been corrected, and minor changes in grammar and style have been made.

Reviewers’ Comments to Revised Submission

Reviewer 1: Jaensch, Robert

Date received: 06-Jul-2023
Reviewer recommendation: **Return to author for minor modifications**
Reviewer overall scoring: **High**

Comments to author:

The article is improved and suggestions have been implemented in the new version.

Still, some language flaws have to be corrected before publication, as well as the style of references 1., 19., 39., 49. and 51 (mainly the year of publication, in 51 the authors are also missing).

Reviewer 2: anonymous

Date received: 06-Jul-2023
Reviewer recommendation: **Accept in present form**
Reviewer overall scoring: **High**

Comments to author:

Appropriately revised manuscript; ready to be published in its present form.

Comments by the Editor-in-Chief Decision on revised version

All reviewer comments were addressed adequately, and the manuscript should be published in its present stage.
